# Supplementary material for: Epidemiology and molecular characterization of Staphylococcus aureus causing bovine mastitis in water buffaloes from the Hazara division of Khyber Pakhtunkhwa, Pakistan
Source: PLoS One. 2022 May 5;17(5):e0268152. doi: 10.1371/journal.pone.0268152 (PMC9071125; doi:10.1371/journal.pone.0268152)
Supplement: S1 Table — (PDF) [file pone.0268152.s001.pdf]

Supplementary Table 1. Summary of the *Staphylococcus aureus* isolated from buffaloes in Hazara Divison

							**Molecular typing**										**Antibiotic Susceptibility**																	
**Sample ID**	**Region**	**Farm**	**Animal**	**Quarter**	**Disease**	**MSSA/MRSA**	***mecA***	***mupA***	***qacA/B***	***smr***	**SCC*mec***	**ACME**	**PVL**	***agr***	***spa* type**	**ST type**	**Ampicillin**	**Cefoxitin**	**Clindamycin**	**Gentamycin**	**Amoxicillin**	**Doxycycline**	**Lincomycin**	**Ceftazidime**	**Rifampin**	**SMX-TMP**	**Linexolid**	**Azithromycin**	**Ceftriaxone**	**Tetracycline**	**Norfloxacin**	**Erythromycin**
BSC28d	Abbottabad	6	28	LR	Sub	MSSA	-	-	-	-	na	-	-	II	t7867	ST9	32 (S)	31 (S)	25 (S)	24 (S)	33 (S)	27 (S)	20 (I)	6 (R)	29 (S)	24 (S)	23 (S)	15 (I)	20 (I)	29 (S)	20 (S)	22 (I)
BSC37b	Abbottabad	8	37	RR	Sub	MSSA	-	-	-	-	na	-	-	II	t7867	ST9	30 (S)	26 (I)	19 (I)	17 (S)	21 (S)	26 (S)	24 (S)	0 (R)	27 (S)	5 (R)	29 (S)	25 (S)	22 (S)	28 (S)	30 (S)	29 (S)
BC45b	Abbottabad	9	45	RR	Clin	MSSA	-	-	-	-	na	-	-	II	t7867	ST9	15 (R)	29 (S)	30 (S)	22 (S)	19 (R)	22 (S)	25 (S)	0 (R)	22 (S)	0 (R)	32 (S)	21 (S)	17 (I)	29 (S)	32 (S)	30 (S)
AC51c	Haripur	11	1	LF	Clin	MSSA	-	-	-	-	na	-	-	II	t7867	ST9	35 (S)	30 (S)	20 (I)	20 (S)	27 (S)	25 (S)	23 (S)	0 (R)	26 (S)	20 (S)	30 (S)	22 (S)	21 (S)	25 (S)	25 (S)	25 (S)
ASC54b	Haripur	11	54	RR	Sub	MSSA	-	-	-	-	na	-	-	II	t7867	ST9	28 (R)	26 (I)	26 (S)	20 (S)	30 (S)	28 (S)	10 (R)	0 (R)	12 (R)	20 (S)	30 (S)	25 (S)	20 (I)	25 (S)	27 (S)	28 (S)
ESC2d	Torghar	1	2	LR		MSSA	-	-	-	-	na	-	-	II	t7867	ST9	35 (S)	30 (S)	32 (S)	30 (S)	39 (S)	30 (S)	17 (I)	0 (R)	17 (I)	25 (S)	32 (S)	27 (S)	15 (I)	30 (S)	27 (S)	25 (S)
FC4c	Kohistan (Upper)	1	4	LF	Clin	MSSA	-	-	-	-	na	-	-	II	t7867	ST9	29 (S)	25 (I)	21 (S)	28 (S)	9 (R)	32 (S)	10 (R)	0 (R)	28 (S)	25 (S)	31 (S)	28 (S)	19 (I)	30 (S)	26 (S)	29 (S)
ASC22b	Haripur	5	22	RR	Sub	MSSA	-	-	-	-	na	-	-	II	t7867	ST9	25 (R)	25 (I)	28 (S)	19 (S)	10 (R)	23 (S)	15 (I)	1 (R)	27 (S)	22 (S)	33 (S)	18 (S)	17 (I)	28 (S)	22 (S)	23 (S)
ASC33c	Haripur	7	33	LF	Sub	MSSA	-	-	-	-	na	-	-	II	t7867	ST9	33 (S)	26 (I)	22 (S)	20 (S)	27 (S)	24 (S)	15 (I)	0 (R)	22 (S)	21 (S)	28 (S)	17 (I)	18 (I)	27 (S)	21 (S)	20 (I)
ASC3c	Haripur	1	3	LF	Sub	MSSA	-	-	-	-	na	-	-	II	t7867	ST9	33 (S)	30 (S)	21 (S)	11 (R)	16 (R)	18 (S)	16 (I)	0 (R)	25 (S)	20 (S)	30 (S)	19 (S)	20 (I)	21 (S)	23 (S)	28 (S)
BC11c	Abbottabad	3	11	LF	Clin	MSSA	-	-	-	-	na	-	-	II	t7867	ST9	31 (S)	25 (I)	26 (S)	19 (S)	30 (S)	23 (S)	22 (S)	8 (R)	25 (S)	20 (S)	25 (S)	20 (S)	20 (I)	25 (S)	27 (S)	25 (S)

BC20d	Abbottabad	4	20	LR	Clin	MSSA	-	-	-	-	na	-	-	II	t7867	ST9	29 (S)	28 (S)	26 (S)	25 (S)	22 (S)	28 (S)	22 (S)	0 (R)	30 (S)	25 (S)	24 (S)	13 (R)	18 (I)	31 (S)	20 (S)	23 (S)
ASC37c	Haripur	8	37	LF	Sub	MSSA	-	-	-	-	na	-	-	II	t7867	ST9	30 (S)	29 (S)	22 (S)	25 (S)	26 (S)	27 (S)	12 (R)	0 (R)	28 (S)	23 (S)	33 (S)	28 (S)	18 (I)	29 (S)	30 (S)	28 (S)
GSC15c	Kohistan (Lower)	3	15	LF	Sub	MSSA	-	-	-	-	na	-	-	II	t7286	ST9	42 (S)	26 (I)	32 (S)	30 (S)	36 (S)	30 (S)	17 (I)	0 (R)	18 (I)	25 (S)	32 (S)	27 (S)	15 (I)	30 (S)	27 (S)	25 (S)
GSC5d	Kohistan (Lower)	1	5	LR	Sub	MSSA	-	-	-	-	na	-	-	II	t7867	ST9	30 (S)	24 (I)	21 (S)	30 (S)	27 (S)	33 (S)	12 (R)	0 (R)	32 (S)	22 (S)	34 (S)	28 (S)	17 (I)	33 (S)	29 (S)	34 (S)
CSC31d	Mansehra	7	31	LR	Sub	MSSA	-	-	-	-	na	-	-	II	t7867	ST9	26 (R)	25 (I)	25 (S)	21 (S)	19 (R)	27 (S)	21 (S)	0 (R)	28 (S)	18 (S)	31 (S)	29 (S)	20 (I)	30 (S)	24 (S)	25 (S)
FSC29c	Kohistan (Upper)	6	29	LF	Sub	MSSA	-	-	-	-	na	-	-	II	t7286	ST9	36 (S)	30 (S)	26 (S)	17 (S)	27 (S)	26 (S)	16 (I)	0 (R)	24 (S)	18 (S)	25 (S)	18 (S)	21 (S)	15 (I)	21 (S)	20 (I)
CSC21d	Mansehra	5	21	LR	Sub	MSSA	-	-	-	-	na	-	-	II	t7867	ST9	35 (S)	26 (I)	20 (I)	18 (S)	24 (S)	20 (S)	7 (R)	1 (R)	20 (S)	18 (S)	30 (S)	20 (S)	26 (S)	25 (S)	20 (S)	18 (I)
FSC48c	Kohistan (Upper)	10	48	LF	Sub	MSSA	-	-	-	-	na	-	-	II	t7867	ST9	15 (R)	25 (I)	26 (S)	22 (S)	25 (S)	27 (S)	22 (S)	7 (R)	23 (S)	18 (S)	32 (S)	21 (S)	22 (S)	32 (S)	25 (S)	27 (S)
ESC26c	Torghar	6	26	LF	Sub	MSSA	-	-	-	-	na	-	-	II	t7867	ST9	40 (S)	31 (S)	25 (S)	20 (S)	36 (S)	25 (S)	20 (I)	0 (R)	25 (S)	20 (S)	30 (S)	20 (S)	20 (I)	25 (S)	27 (S)	25 (S)
CC27d	Mansehra	6	27	LR	Clin	MSSA	-	-	-	-	na	-	-	II	t7867	ST9	33 (S)	26 (I)	26 (S)	16 (S)	22 (S)	26 (S)	16 (I)	0 (R)	24 (S)	18 (S)	25 (S)	18 (S)	21 (S)	9 (R)	21 (S)	21 (I)
CSC33d	Mansehra	7	33	LR	Sub	MSSA	-	-	-	-	na	-	-	II	t7867	ST9	35 (S)	24 (I)	23 (S)	20 (S)	25 (S)	25 (S)	18 (I)	7 (R)	28 (S)	25 (S)	30 (S)	14 (I)	24 (S)	25 (S)	20 (S)	30 (S)
DSC1d	Battagram	1	1	LR	Sub	MSSA	-	-	-	-	na	-	-	II	t7867	ST9	28 (R)	24 (I)	21 (S)	23 (S)	20 (S)	27 (S)	18 (I)	0 (R)	28 (S)	21 (S)	30 (S)	24 (S)	18 (I)	24 (S)	26 (S)	27 (S)
ESC40d	Torghar	8	40	LR	Sub	MSSA	-	-	-	-	na	-	-	II	t7867	ST9	15 (R)	28 (S)	19 (I)	17 (S)	20 (S)	26 (S)	24 (S)	0 (R)	27 (S)	5 (R)	29 (S)	25 (S)	22 (S)	28 (S)	30 (S)	29 (S)
AC31a	Haripur	7	31	RF	Clin	MSSA	-	-	-	-	na	-	-	II	t7286	ST9	33 (S)	28 (S)	25 (S)	28 (S)	26 (S)	30 (S)	17 (I)	9 (R)	27 (S)	23 (S)	30 (S)	26 (S)	22 (S)	25 (S)	28 (S)	28 (S)
CSC24d	Mansehra	5	24	LR	Sub	MSSA	-	-	-	-	na	-	-	II	t7867	ST9	30 (S)	27 (I)	20 (I)	20 (S)	27 (S)	24 (S)	20 (I)	0 (R)	30 (S)	22 (S)	33 (S)	20 (S)	26 (S)	30 (S)	20 (S)	25 (S)

BC45c	Abbottabad	9	45	LF	Clin	MSSA	-	-	-	-	na	-	-	II	t7286	ST9	29 (S)	30 (S)	30 (S)	22 (S)	12 (R)	24 (S)	26 (S)	0 (R)	20 (S)	15 (I)	30 (S)	20 (S)	20 (I)	28 (S)	30 (S)	25 (S)
ESC11a	Torghar	3	11	RF	Sub	MSSA	-	-	-	-	na	-	-	II	t7286	ST9	6 (R)	25 (I)	26 (S)	20 (S)	29 (S)	28 (S)	10 (R)	0 (R)	6 (R)	20 (S)	30 (S)	25 (S)	20 (I)	25 (S)	27 (S)	28 (S)
CSC31c	Mansehra	7	31	LF	Sub	MSSA	-	-	-	-	na	-	-	II	t7867	ST9	37 (S)	30 (S)	26 (S)	16 (S)	29 (S)	26 (S)	16 (I)	0 (R)	24 (S)	18 (S)	25 (S)	18 (S)	21 (S)	29 (S)	21 (S)	21 (I)
FC40a	Kohistan (Upper)	8	40	RF	Clin	MSSA	-	-	-	-	na	-	-	II	t7867	ST9	35 (S)	25 (I)	23 (S)	20 (S)	23 (S)	25 (S)	18 (I)	0 (R)	28 (S)	25 (S)	30 (S)	14 (I)	24 (S)	25 (S)	20 (S)	30 (S)
FSC37b	Kohistan (Upper)	8	37	RR	Sub	MSSA	-	-	-	-	na	-	-	II	t7867	ST9	28 (R)	25 (I)	25 (S)	21 (S)	11 (R)	27 (S)	21 (S)	6 (R)	28 (S)	18 (S)	31 (S)	29 (S)	20 (I)	30 (S)	24 (S)	25 (S)
EC17d	Torghar	4	17	LR	Clin	MSSA	-	-	-	-	na	-	-	II	t7867	ST9	38 (S)	27 (I)	26 (S)	19 (S)	34 (S)	23 (S)	22 (S)	8 (R)	25 (S)	20 (S)	25 (S)	20 (S)	20 (I)	25 (S)	27 (S)	25 (S)
FSC10c	Kohistan (Upper)	2	10	LF	Sub	MSSA	-	-	-	-	na	-	-	II	t7286	ST9	37 (S)	24 (I)	26 (S)	26 (S)	23 (S)	27 (S)	8 (R)	0 (R)	27 (S)	7 (R)	32 (S)	27 (S)	20 (I)	27 (S)	30 (S)	27 (S)
GC53c	Kohistan (Lower)	11	53	LF	Clin	MSSA	-	-	-	-	na	-	-	II	t7867	ST9	35 (S)	25 (I)	25 (S)	23 (S)	23 (S)	23 (S)	13 (R)	5 (R)	28 (S)	22 (S)	28 (S)	22 (S)	21 (S)	25 (S)	25 (S)	18 (I)
CSC48c	Mansehra	10	48	LF	Sub	MSSA	-	-	-	-	na	-	-	II	t7286	ST9	34 (S)	29 (S)	26 (S)	22 (S)	26 (S)	27 (S)	22 (S)	0 (R)	23 (S)	18 (S)	32 (S)	21 (S)	22 (S)	32 (S)	25 (S)	27 (S)
GC12d	Kohistan (Lower)	3	12	LR	Clin	MSSA	-	-	-	-	na	-	-	II	t7286	ST9	35 (S)	32 (S)	20 (I)	20 (S)	33 (S)	25 (S)	23 (S)	10 (R)	26 (S)	20 (S)	30 (S)	22 (S)	21 (S)	25 (S)	25 (S)	25 (S)
CSC16a	Mansehra	4	16	RF	Sub	MSSA	-	-	-	-	na	-	-	II	t7286	ST9	26 (R)	24 (I)	26 (S)	22 (S)	29 (S)	26 (S)	29 (S)	0 (R)	26 (S)	23 (S)	30 (S)	24 (S)	19 (I)	25 (S)	20 (S)	25 (S)
AC43c	Haripur	9	43	LF	Clin	MSSA	-	-	-	-	na	-	-	II	t7286	ST9	32 (S)	28 (S)	27 (S)	26 (S)	12 (R)	27 (S)	23 (S)	4 (R)	28 (S)	24 (S)	29 (S)	25 (S)	17 (I)	27 (S)	25 (S)	25 (S)
FSC15a	Kohistan (Upper)	3	15	RF	Sub	MSSA	-	-	-	-	na	-	-	II	t7286	ST9	30 (S)	27 (I)	26 (S)	22 (S)	25 (S)	26 (S)	29 (S)	7 (R)	26 (S)	23 (S)	30 (S)	24 (S)	19 (I)	25 (S)	20 (S)	25 (S)
EC55d	Torghar	11	55	LR	Clin	MSSA	-	-	-	-	na	-	-	II	t7867	ST9	29 (S)	26 (I)	30 (S)	22 (S)	27 (S)	22 (S)	25 (S)	7 (R)	22 (S)	0 (R)	32 (S)	21 (S)	17 (I)	29 (S)	32 (S)	30 (S)
FC40d	Kohistan (Upper)	8	40	LR	Clin	MSSA	-	-	-	-	na	-	-	II	t7867	ST9	25 (R)	24 (I)	27 (S)	21 (S)	32 (S)	26 (S)	19 (I)	0 (R)	27 (S)	19 (S)	31 (S)	21 (S)	23 (S)	27 (S)	23 (S)	23 (S)

AC17a	Haripur	4	17	RF	Clin	MSSA	-	-	-	-	na	-	-	II	t7286	ST9	30 (S)	25 (I)	20 (I)	24 (S)	19 (R)	28 (S)	6 (R)	0 (R)	29 (S)	22 (S)	30 (S)	25 (S)	17 (I)	27 (S)	25 (S)	25 (S)
DSC31c	Battagram	7	31	LF	Sub	MSSA	-	-	-	-	na	-	-	II	t7867	ST9	33 (S)	29 (S)	25 (S)	28 (S)	25 (S)	30 (S)	17 (I)	9 (R)	27 (S)	23 (S)	30 (S)	26 (S)	22 (S)	25 (S)	28 (S)	28 (S)
DSC34b	Battagram	7	34	RR	Sub	MSSA	-	-	-	-	na	-	-	II	t7867	ST9	23 (R)	24 (I)	23 (S)	24 (S)	23 (S)	22 (S)	16 (I)	0 (R)	26 (S)	19 (S)	28 (S)	25 (S)	21 (S)	26 (S)	27 (S)	25 (S)
ESC28a	Torghar	6	28	RF	Sub	MSSA	-	-	-	-	na	-	-	II	t7867	ST9	28 (R)	28 (S)	25 (S)	24 (S)	31 (S)	27 (S)	20 (I)	0 (R)	29 (S)	24 (S)	23 (S)	15 (I)	20 (I)	29 (S)	20 (S)	21 (I)
CSC52b	Mansehra	11	52	RR	Sub	MSSA	-	-	-	-	na	-	-	II	t7867	ST9	32 (S)	33 (S)	21 (S)	10 (R)	0 (R)	18 (S)	16 (I)	0 (R)	25 (S)	20 (S)	30 (S)	19 (S)	20 (I)	21 (S)	23 (S)	28 (S)
FSC9c	Kohistan (Upper)	2	9	LF	Sub	MSSA	-	-	-	-	na	-	-	II	t7867	ST9	33 (S)	25 (I)	25 (S)	25 (S)	33 (S)	23 (S)	13 (R)	5 (R)	28 (S)	22 (S)	28 (S)	22 (S)	21 (S)	25 (S)	25 (S)	20 (I)
DC15c	Battagram	3	15	LF	Clin	MSSA	-	-	-	-	na	-	-	II	t7867	ST9	39 (S)	30 (S)	33 (S)	21 (S)	32 (S)	35 (S)	25 (S)	3 (R)	33 (S)	28 (S)	30 (S)	20 (S)	25 (S)	32 (S)	28 (S)	29 (S)
FSC23c	Kohistan (Upper)	5	23	LF	Sub	MSSA	-	-	-	-	na	-	-	II	t7867	ST9	34 (S)	27 (I)	20 (I)	20 (S)	29 (S)	24 (S)	20 (I)	0 (R)	30 (S)	22 (S)	33 (S)	20 (S)	26 (S)	30 (S)	20 (S)	25 (S)
GSC47d	Kohistan (Lower)	10	47	LR	Sub	MSSA	-	-	-	-	na	-	-	II	t7286	ST9	39 (S)	29 (S)	21 (S)	28 (S)	13 (R)	32 (S)	10 (R)	0 (R)	28 (S)	25 (S)	31 (S)	28 (S)	19 (I)	30 (S)	26 (S)	29 (S)
AC17c	Haripur	4	17	LF	Clin	MSSA	-	-	-	-	na	-	-	II	t7286	ST9	28 (R)	28 (S)	21 (S)	23 (S)	25 (S)	27 (S)	18 (I)	0 (R)	28 (S)	21 (S)	30 (S)	24 (S)	18 (I)	24 (S)	26 (S)	27 (S)
AC31b	Haripur	7	31	RR	Clin	MSSA	-	-	-	-	na	-	-	II	t7286	ST9	23 (R)	25 (I)	23 (S)	24 (S)	20 (S)	22 (S)	16 (I)	0 (R)	26 (S)	19 (S)	28 (S)	25 (S)	21 (S)	26 (S)	27 (S)	25 (S)
FSC2a	Kohistan (Upper)	1	2	RF	Sub	MSSA	-	-	-	-	na	-	-	II	t7286	ST9	36 (S)	29 (S)	30 (S)	22 (S)	32 (S)	24 (S)	26 (S)	0 (R)	20 (S)	15 (I)	30 (S)	20 (S)	20 (I)	28 (S)	30 (S)	25 (S)
ASC48a	Haripur	10	48	RF	Sub	MSSA	-	-	-	-	na	-	-	II	t7286	ST9	30 (S)	24 (I)	21 (S)	31 (S)	20 (S)	33 (S)	12 (R)	0 (R)	32 (S)	22 (S)	34 (S)	28 (S)	17 (I)	33 (S)	29 (S)	34 (S)
FSC53a	Kohistan (Upper)	11	53	RF	Sub	MSSA	-	-	-	-	na	-	-	II	t7286	ST9	33 (S)	29 (S)	22 (S)	25 (S)	24 (S)	27 (S)	12 (R)	0 (R)	28 (S)	23 (S)	33 (S)	28 (S)	18 (I)	9 (R)	30 (S)	28 (S)
ASC22c	Haripur	5	22	LF	Sub	MSSA	-	-	-	-	na	-	-	II	t7286	ST9	35 (S)	31 (S)	28 (S)	20 (S)	28 (S)	25 (S)	10 (R)	7 (R)	33 (S)	25 (S)	19 (R)	21 (S)	22 (S)	29 (S)	20 (S)	24 (S)

CSC19a	Mansehra	4	19	RF	Sub	MSSA	-	-	-	-	na	-	-	II	t7286	ST9	34 (S)	32 (S)	25 (S)	22 (S)	23 (S)	27 (S)	22 (S)	0 (R)	29 (S)	23 (S)	31 (S)	17 (I)	19 (I)	30 (S)	18 (I)	25 (S)
FSC48d	Kohistan (Upper)	10	48	LR	Sub	MSSA	-	-	-	-	na	-	-	II	t7286	ST9	22 (R)	30 (S)	22 (S)	20 (S)	19 (R)	24 (S)	15 (I)	0 (R)	22 (S)	21 (S)	28 (S)	16 (I)	18 (I)	27 (S)	21 (S)	21 (I)
DSC51c	Battagram	11	51	LF	Sub	MSSA	-	-	-	-	na	-	-	II	t7286	ST9	28 (R)	25 (I)	20 (I)	20 (S)	10 (R)	25 (S)	23 (S)	0 (R)	26 (S)	20 (S)	30 (S)	22 (S)	21 (S)	25 (S)	25 (S)	25 (S)
GSC15d	Kohistan (Lower)	3	15	LR	Sub	MSSA	-	-	-	-	na	-	-	II	t7286	ST9	36 (S)	29 (S)	26 (S)	20 (S)	30 (S)	28 (S)	10 (R)	0 (R)	10 (R)	20 (S)	30 (S)	25 (S)	20 (I)	25 (S)	27 (S)	28 (S)
DSC45d	Battagram	9	45	LR	Sub	MSSA	-	-	-	-	na	-	-	II	t7286	ST9	30 (S)	24 (I)	21 (S)	31 (S)	33 (S)	33 (S)	12 (R)	8 (R)	32 (S)	22 (S)	34 (S)	28 (S)	17 (I)	33 (S)	29 (S)	34 (S)
FSC14c	Kohistan (Upper)	3	14	LF	Sub	MSSA	-	-	-	-	na	-	-	II	t7867	ST9	16 (R)	28 (S)	24 (S)	24 (S)	21 (S)	27 (S)	14 (R)	0 (R)	26 (S)	14 (I)	31 (S)	25 (S)	28 (S)	24 (S)	31 (S)	30 (S)
GSC5b	Kohistan (Lower)	1	5	RR	Sub	MSSA	-	-	-	-	na	-	-	II	t7867	ST9	32 (S)	31 (S)	27 (S)	26 (S)	26 (S)	27 (S)	23 (S)	0 (R)	28 (S)	24 (S)	29 (S)	25 (S)	17 (I)	27 (S)	25 (S)	25 (S)
FSC33a	Kohistan (Upper)	7	33	RF	Sub	MSSA	-	-	-	-	na	-	-	II	t7867	ST9	31 (S)	28 (S)	26 (S)	21 (S)	17 (R)	26 (S)	16 (I)	0 (R)	24 (S)	18 (S)	25 (S)	18 (S)	21 (S)	29 (S)	21 (S)	16 (I)
DC43b	Battagram	9	43	RR	Clin	MSSA	-	-	-	-	na	-	-	II	t7867	ST9	29 (S)	26 (I)	22 (S)	25 (S)	23 (S)	27 (S)	12 (R)	6 (R)	28 (S)	23 (S)	33 (S)	28 (S)	18 (I)	29 (S)	30 (S)	28 (S)
HSC21d	Kolai Palas	5	21	LR	Sub	MSSA	-	-	-	-	na	-	-	II	t7867	ST9	16 (R)	29 (S)	24 (S)	25 (S)	0 (R)	27 (S)	14 (R)	8 (R)	26 (S)	14 (I)	31 (S)	25 (S)	28 (S)	24 (S)	31 (S)	30 (S)
BSC47c	Abbottabad	10	47	LF	Sub	MSSA	-	-	-	-	na	-	-	II	t7286	ST9	29 (S)	27 (I)	21 (S)	28 (S)	13 (R)	32 (S)	10 (R)	0 (R)	28 (S)	25 (S)	31 (S)	28 (S)	19 (I)	30 (S)	26 (S)	29 (S)
CSC35d	Mansehra	7	35	LR	Sub	MSSA	-	-	-	-	na	-	-	II	t7867	ST9	32 (S)	27 (I)	27 (S)	21 (S)	11 (R)	26 (S)	19 (I)	0 (R)	27 (S)	19 (S)	31 (S)	21 (S)	23 (S)	27 (S)	23 (S)	23 (S)
DSC34d	Battagram	7	34	LR	Sub	MSSA	-	-	-	-	na	-	-	II	t7867	ST9	22 (R)	28 (S)	22 (S)	20 (S)	27 (S)	24 (S)	15 (I)	0 (R)	22 (S)	21 (S)	28 (S)	16 (I)	18 (I)	27 (S)	21 (S)	16 (I)
BSC51b	Abbottabad	11	51								na						25 (R)	25 (I)	25 (S)	23 (S)	28 (S)	23 (S)	13 (R)	5 (R)	28 (S)	22 (S)	28 (S)	22 (S)	21 (S)	25 (S)	25 (S)	19 (I)
EC31c	Torghar	7	31	LF	Clin	MSSA	-	-	-	-	na	-	-	I	t2078	ST101	30 (S)	28 (S)	27 (S)	25 (S)	28 (S)	30 (S)	24 (S)	10 (R)	31 (S)	23 (S)	28 (S)	5 (R)	8 (R)	30 (S)	21 (S)	18 (I)

FC17d	Kohistan (Upper)	4	17	LR	Clin	MSSA	-	-	-	-	na	-	-	I	t2078	ST101	39 (S)	26 (I)	20 (I)	19 (S)	31 (S)	20 (S)	7 (R)	1 (R)	17 (I)	18 (S)	30 (S)	20 (S)	26 (S)	25 (S)	20 (S)	17 (I)
CC55c	Mansehra	11	55	LF	Clin	MSSA	-	-	-	-	na	-	-	I	t2078	ST101	28 (R)	30 (S)	20 (I)	22 (S)	30 (S)	31 (S)	17 (I)	10 (R)	27 (S)	21 (S)	30 (S)	20 (S)	25 (S)	31 (S)	28 (S)	26 (S)
FC17a	Kohistan (Upper)	4	17	RF	Clin	MSSA	-	-	-	-	na	-	-	I	t2078	ST101	32 (S)	33 (S)	25 (S)	22 (S)	20 (S)	27 (S)	22 (S)	0 (R)	29 (S)	23 (S)	31 (S)	17 (I)	19 (I)	30 (S)	18 (I)	25 (S)
BC11d	Abbottabad	3	11	LR	Clin	MSSA	-	-	-	-	na	-	-	I	t2078	ST101	36 (S)	27 (I)	25 (S)	20 (S)	28 (S)	25 (S)	20 (I)	0 (R)	25 (S)	20 (S)	30 (S)	20 (S)	20 (I)	25 (S)	27 (S)	25 (S)
BSC13d	Abbottabad	3	13	LR	Sub	MSSA	-	-	-	-	na	-	-	I	t2078	ST101	27 (R)	24 (I)	25 (S)	24 (S)	13 (R)	27 (S)	20 (I)	0 (R)	29 (S)	24 (S)	23 (S)	15 (I)	20 (I)	29 (S)	20 (S)	22 (I)
BSC41a	Abbottabad	9	41	RF	Sub	MSSA	-	-	-	-	na	-	-	I	t2078	ST101	36 (S)	31 (S)	35 (S)	25 (S)	26 (S)	30 (S)	30 (S)	7 (R)	29 (S)	6 (R)	33 (S)	29 (S)	25 (S)	16 (I)	32 (S)	30 (S)
DC43c	Battagram	9	43	LF	Clin	MSSA	-	-	-	-	na	-	-	I	t2078	ST101	32 (S)	35 (S)	27 (S)	26 (S)	31 (S)	27 (S)	23 (S)	0 (R)	28 (S)	24 (S)	29 (S)	25 (S)	17 (I)	27 (S)	25 (S)	25 (S)
EC17b	Torghar	4	17	RR	Clin	MSSA	-	-	-	-	na	-	-	I	t2078	ST101	25 (R)	25 (I)	27 (S)	21 (S)	21 (S)	26 (S)	19 (I)	0 (R)	29 (S)	19 (S)	31 (S)	21 (S)	23 (S)	27 (S)	23 (S)	23 (S)
BSC37c	Abbottabad	8	37	LF	Sub	MSSA	-	-	-	-	na	-	-	I	t2078	ST101	33 (S)	29 (S)	25 (S)	19 (S)	27 (S)	25 (S)	17 (I)	0 (R)	23 (S)	9 (R)	29 (S)	20 (S)	20 (I)	28 (S)	25 (S)	9 (R)
CC55d	Mansehra	11	55	LR	Clin	MSSA	-	-	-	-	na	-	-	I	t2078	ST101	30 (S)	25 (I)	20 (I)	24 (S)	7 (R)	28 (S)	6 (R)	0 (R)	29 (S)	22 (S)	30 (S)	25 (S)	17 (I)	27 (S)	25 (S)	25 (S)
ESC34d	Torghar	7	34	LR	Sub	MSSA	-	-	-	-	na	-	-	I	t2078	ST101	21 (R)	24 (I)	26 (S)	25 (S)	22 (S)	28 (S)	22 (S)	0 (R)	30 (S)	25 (S)	24 (S)	10 (R)	18 (I)	31 (S)	20 (S)	23 (S)
DSC19b	Battagram	4	19	RR	Sub	MRSA	+	-	-	-	IVa	-	-	I	t8934	ST22	21 (R)	13 (R)	28 (S)	19 (S)	10 (R)	23 (S)	15 (I)	1 (R)	27 (S)	22 (S)	33 (S)	10 (R)	17 (I)	28 (S)	22 (S)	23 (S)
HC12d	Kolai Palas	3	12	LR		MRSA	+	-	-	-	IVa	-	-	I	t8934	ST22	15 (R)	11 (R)	26 (S)	26 (S)	21 (S)	10 (R)	8 (R)	0 (R)	27 (S)	7 (R)	14 (R)	27 (S)	20 (I)	27 (S)	30 (S)	10 (R)
ESC37b	Torghar	8	37	RR	Sub	MRSA	+	-	-	-	IVa	-	-	I	t8934	ST22	24 (R)	16 (R)	25 (S)	24 (S)	21 (S)	27 (S)	20 (I)	0 (R)	29 (S)	24 (S)	23 (S)	15 (I)	20 (I)	29 (S)	20 (S)	21 (I)
ESC45c	Torghar	9	45	LF	Sub	MRSA	+	-	-	-	IVa	-	-	I	t8934	ST22	9 (R)	17 (R)	25 (S)	19 (S)	30 (S)	25 (S)	17 (I)	0 (R)	23 (S)	9 (R)	29 (S)	20 (S)	20 (I)	18 (I)	25 (S)	22 (I)

ESC11c	Torghar	3	11	LF	Sub	MRSA	+	-	-	-	IVa	-	-	I	t8934	ST22	12 (R)	15 (R)	23 (S)	18 (S)	19 (R)	25 (S)	22 (S)	5 (R)	26 (S)	17 (S)	23 (S)	20 (S)	18 (I)	22 (S)	23 (S)	23 (S)
GSC42d	Kohistan (Lower)	9	42	LR	Sub	MRSA	+	-	-	-	IVa	-	-	I	t8934	ST22	16 (R)	15 (R)	30 (S)	22 (S)	24 (S)	24 (S)	26 (S)	0 (R)	20 (S)	15 (I)	30 (S)	20 (S)	20 (I)	8 (R)	30 (S)	25 (S)
GSC21d	Kohistan (Lower)	5	21	LR	Sub	MRSA	+	-	-	-	IVa	-	-	I	t8934	ST22	29 (S)	20 (R)	35 (S)	25 (S)	20 (S)	30 (S)	30 (S)	0 (R)	29 (S)	6 (R)	33 (S)	29 (S)	11 (R)	30 (S)	32 (S)	11 (R)
GSC31d	Kohistan (Lower)	7	31	LR	Sub	MRSA	+	-	-	-	IVa	-	-	I	t8934	ST22	15 (R)	18 (R)	30 (S)	29 (S)	0 (R)	22 (S)	25 (S)	4 (R)	22 (S)	0 (R)	32 (S)	21 (S)	17 (I)	29 (S)	32 (S)	30 (S)
DSC27a	Battagram	6	27	RF	Sub	MRSA	+	-	-	-	IVa	-	-	I	t8934	ST22	23 (R)	8 (R)	28 (S)	20 (S)	15 (R)	9 (R)	18 (I)	10 (R)	29 (S)	19 (S)	25 (S)	6 (R)	27 (S)	24 (S)	20 (S)	29 (S)
BC20c	Abbottabad	4	20	LF	Clin	MRSA	+	-	-	-	IVa	-	-	I	t8934	ST22	20 (R)	11 (R)	27 (S)	25 (S)	8 (R)	30 (S)	24 (S)	9 (R)	31 (S)	23 (S)	28 (S)	8 (R)	12 (R)	30 (S)	21 (S)	18 (I)
DSC25c	Battagram	5	25	LF	Sub	MRSA	+	-	-	-	IVa	-	-	I	t8934	ST22	17 (R)	15 (R)	28 (S)	20 (S)	17 (R)	25 (S)	10 (R)	7 (R)	33 (S)	25 (S)	20 (R)	21 (S)	7 (R)	12 (R)	20 (S)	24 (S)
AC17d	Haripur	4	17	LR	Clin	MRSA	+	-	-	-	IVa	-	-	I	t8934	ST22	26 (R)	16 (R)	33 (S)	21 (S)	22 (S)	35 (S)	25 (S)	3 (R)	33 (S)	28 (S)	30 (S)	20 (S)	25 (S)	32 (S)	28 (S)	29 (S)
ESC50a	Torghar	10	50	RF	Sub	MRSA	+	-	-	-	IVa	-	-	I	t8934	ST22	27 (R)	12 (R)	35 (S)	25 (S)	22 (S)	30 (S)	30 (S)	0 (R)	29 (S)	6 (R)	33 (S)	29 (S)	5 (R)	30 (S)	32 (S)	30 (S)
ASC7d	Haripur	2	7	LR	Clin	MRSA	+	-	-	-	IVa	-	-	I	t8934	ST22	24 (R)	12 (R)	20 (I)	20 (S)	22 (S)	31 (S)	17 (I)	0 (R)	27 (S)	21 (S)	30 (S)	9 (R)	25 (S)	8 (R)	28 (S)	26 (S)
AC51d	Haripur	11	51	LR	Clin	MRSA	+	-	-	-	IVa	-	-	I	t8934	ST22	25 (R)	10 (R)	32 (S)	30 (S)	10 (R)	30 (S)	17 (I)	10 (R)	19 (I)	25 (S)	32 (S)	27 (S)	15 (I)	30 (S)	27 (S)	25 (S)
CSC10d	Mansehra	2	10	LR	Sub	MRSA	+	-	-	-	IVa	-	-	I	t8934	ST22	16 (R)	9 (R)	24 (S)	24 (S)	0 (R)	27 (S)	14 (R)	8 (R)	26 (S)	14 (I)	31 (S)	25 (S)	28 (S)	17 (I)	31 (S)	30 (S)
CSC3b	Mansehra	1	3	RR	Sub	MRSA	+	-	-	-	IVa	-	-	I	t8934	ST22	15 (R)	7 (R)	26 (S)	26 (S)	20 (S)	27 (S)	8 (R)	0 (R)	27 (S)	7 (R)	32 (S)	27 (S)	20 (I)	27 (S)	30 (S)	27 (S)
BSC2c	Abbottabad	1	2	LF	Sub	MRSA	+	-	-	-	IVa	-	-	I	t8934	ST22	12 (R)	15 (R)	23 (S)	18 (S)	0 (R)	25 (S)	22 (S)	0 (R)	26 (S)	17 (S)	23 (S)	20 (S)	18 (I)	22 (S)	23 (S)	23 (S)
BSC2d	Abbottabad	1	2	LR	Sub	MRSA	+	-	-	-	IVa	-	-	I	t8934	ST22	25 (R)	17 (R)	27 (S)	21 (S)	11 (R)	26 (S)	19 (I)	0 (R)	27 (S)	19 (S)	31 (S)	21 (S)	10 (R)	11 (R)	23 (S)	23 (S)

ASC28c	Haripur	6	28	LF	Sub	MRSA	+	-	-	-	IVa	-	-	I	t8934	ST22	29 (S)	9 (R)	28 (S)	20 (S)	15 (R)	5 (R)	18 (I)	10 (R)	29 (S)	19 (S)	25 (S)	11 (R)	6 (R)	24 (S)	20 (S)	29 (S)

Note: *mecA*, methicillin resistance protein; *mupA*, mupirocin resistance protein; *qacA/B*, multidrug efflux pump; *smr*, multidrug resistance protein family; ACME, Arginine Catabolic Membrane Protein; PVL, Panton-Valentine Leukocidin; *agr*, accessory gene reglator; *spa*, staphylococcal protein A; ST, multilocus sequence type; SMX-TMP, Sulfamethoxazole-trimethoprim; FR, front right; FL, front left; RR, rear right; RL, rear left; sub, subclinical infection; clin, clinical infection; -, negative; +, positive; (S), susceptible; (R), resistant; (I), Intermediate resistant.
