# Supplementary material for: Epidemiology and molecular characterization of Staphylococcus aureus causing bovine mastitis in water buffaloes from the Hazara division of Khyber Pakhtunkhwa, Pakistan
Source: PLoS One. 2022 May 5;17(5):e0268152. doi: 10.1371/journal.pone.0268152 (PMC9071125; doi:10.1371/journal.pone.0268152)
Supplement: Attachment — Submitted filename: Response to reviewers.pdf [file pone.0268152.s002.pdf]

Re: PONE-D-22-02764 **Response to reviewers**

Reviewer #1:

Javed et al described the epidemiology and the prevalence of staphylococci causing bovine mastitis in water buffaloes in Hazara division of Pakistan. The authors used PFGE to assess the genetic relatedness of the S. aureus isolates recovered in the study and assessed them for antibiotic resistance. The study is well designed and should add to the growing data and knowledge of MRSA in food production animals in the region Below are some comments for consideration

Minor comments

CoNS was not defined anywhere in the manuscript

-Our apologies, this was a formatting error. The section discussing CoNS was not supposed to be included in the manuscript and failed to get deleted. It has now been removed.

Except when starting a sentence, “staphylococci” or “staphylococcal” should not be written as “Staphylococci”

-The errors have been corrected.

Authors should consider reviewing the punctuations the manuscript.

-A review was done and changes made.

Major comments

1. The introduction is well written and very easy to read. It gave a detailed background of the study and identified the knowledge gaps this study aimed to fill. I would not want to assume that there is no single study from Pakistan that report the molecular characterization either from human, animals, or environmental matrices. Literature on the population structure of S. aureus should be reviewed and included in this section.

-Sorry, you are correct and the sentence was worded poorly. It has been changed to say:

“While numerous studies have examined the prevalence and epidemiology of mastitis in various regions of Pakistan, detailed molecular typing of the staphylococci from cases of mastitis in the country is limited.”

The population structure of *S. aureus* in humans in the region is presented (and has been expanded slightly) in the discussion, as it relates to the results. It truly is very limited with a couple studies only going as far as detecting the *mecA* gene. We feel that adding it to the introduction, specifically at the point where we are presenting the purpose and goals of the paper, would be distracting and dilute out the purpose of the paper. But we agree that the information deserves to be addressed in the paper.

2. The sampling was robust as authors sampled 5 buffaloes in 11 farms from 35 districts but it's not so clear how the farms or the number of buffaloes to be sampled from each farm was determined. If this was a convenience/random sampling, the authors should state this in the manuscript or better still give details on their calculation of the sample size.

-The farms were randomly selected with the criteria that they be at least 5 Km apart and contain at least 5 animals. Animals were conveniently/randomly selected for sampling. This has been added to the materials and methods. A reference was included for how sampling (including numbers) was done.

3. The authors characterized the S. aureus using PFGE and analyzed the results using BioNumerics with a position tolerance of 1.5 as well as an optimization of 0. Are these parameters previously described? If yes, the author should cite appropriate references and if otherwise, a justification for using these parameters should be stated in the manuscript.

-The reference is the same as the one describing the standardized protocol for PFGE typing *S. aureus*. A brief description was added to the section.

4. The authors described a high prevalence of staphylococci in presumably infected milk samples. How many samples carried both S. aureus and other staphylococci or non-staphylococci. Is there co-occurrence of the bacteria in different samples? If yes, is the co-occurrence specific, common or differ among water buffaloes from different districts? What are the identities of the non-staphylococcal isolates described in this study? As I understood, all the isolates are Gram positive and ferment mannitol as well.

-The number of animals co-infected with SA and CoNS has been added to the manuscript and Figure 1. Co-infection with other species was not specifically tracked since only species that grew on our selective media would be accounted for, creating an inaccurate number.

5. The paragraph that addressed molecular characterization in the methods section is quite difficult to follow. The authors should consider splitting this paragraph into two. One for PCR-based and the other for PFGE. In the latter, the authors should consider stating the conditions and other information important for reproducing the results.

-The information was presented in the current order because it provides the easiest workflow for anyone wanting to repeat the experiments. The multiplex PCR is routinely performed first for a quick identification of species and methicillin resistance. PFGE is done next on all *S. aureus* to identify how many strain types/groups are likely present. Antiseptic resistance PCR, SCC*mec* typing, *agr* typing, spa typing and MLST typing are subsequently done for greater detail and strain molecular characterization. Grouping them based on PCR vs PFGE would create a workflow that isn't ideal. In terms of the PFGE conditions, they are described in the references, however the following information was added:

"*S. aureus* isolates were subsequently fingerprinted using pulsed field gel electrophoresis (PFGE) following digestion by SmaI, using a standardized protocols [11]. Briefly, S. aureus agarose plugs were digested with smaI and loaded onto a 1% agarose gel, then electrophoresis done with a CHEF mapper using switch times of 5.3 to 35 for 18 hours at 14°C, 6.0V/cm with an angle of 120 in 0.5x TBE."

6. The authors stated that PCR assay that differentiates S. aureus from coagulase negative staphylococci, while also distinguishing MRSA from MSSA was used. The authors should consider giving a brief detail about this method and the basis for the differentiation, stating the exact gene(s) targeted by the assay. Although one could assume that nuc and mecA genes are targeted but this should be stated in the manuscript. How was the MLST determined?

-Detailed information is available in the reference however a brief mention of the genes used in the multiplex has been added to the text. We apologize about the MLST omission (formatting error again), the reference was added back.

7. The authors reported that the left quarters were twice prone to infection than those on the right side. Can the comment on this observation and its significance to the study? Again, is this district or farm associated?

-The topic was addressed in lines 331-346 of the discussion (original submission), with a slight edit in this version. There is no association between district or farm, which was added to the discussion section.

“We observed that left quarters (both front and rear) had more than twice the infection rate (69.6%) of right quarters (30.4%), which held true in all districts and on all farms.”

8. t8934-ST22-IVa MRSA-IVa was isolated from all the districts except upper Kohistan. What is the prevalence of this clone in health care and community settings in the division studied? Has this clone been reported in animals/livestock or humans in the region? This information is important to contextualize this study.

-This was addressed in the discussion in lines 369-370 (original submission). With such limited molecular data available for Pakistan, the actual prevalence of ST22-MRSA in health care and community settings is unknown, both for the country as a whole and division studied. There was, likewise, no data from other animals/livestock.

“In one study CC22-MRSA-IV was identified in 13.6% (belonging to three different strains) of the human isolates [33], matching the ST type of all the MRSA in our study.”

9. There were also instances of two animals carrying two different clones (ST9-t7867-MSSA and ST101-t2078-MSSA), (ST9-t7867-MSSA and ST22-t8934-MRSA-IVa), (ST9-t7867-MSSA and ST9-MSSA) etc. Are these animals from the same farm? Also, were the two strains recovered from the same quarter? This information is also important.

-No they did not occur in the same farm, as previously stated in the results on lines 247-248 (original submission). They were not in the same quarter either, so the sentence has been amended to read:

“Interestingly, there were ten instances where different strain types were isolated from a single animal, with each strain isolated from different quarters within the animal, and all occurring on different farms.”

10. The authors reported that there was no link between antibiotic resistance and the MSSA strain type, region, or farms. Is there a link between antibiotic resistance and MRSA and the farms or regions?

- We added that MRSA showed no link either. Now read:

“There wasn’t a link between antibiotic resistance and the MSSA/MRSA strain type, region or farm from which the samples came (details available in **S1 Table**).”

11. In the discussion section, the authors stated that studies on staphylococcal strains causing buffalo mastitis within Pakistan is scarce or not available. Are there studies from other food producing animals on this subject?

-Unfortunately we found limited studies with detailed molecular data for *S. aureus* in other food or companion animals. We included what we could find in the discussion. These are examples of the level of detail typically reported in the region.

https://doi.org/10.1089/fpd.2018.2585: PFGE but not shown, mecA, mecC, antibiotic resistance in eggs of household chickens.

https://doi.org/10.3389/fmicb.2020.577707: detected mecA and *spa* amplicon size (not sequenced). In chickens, beef, mutton of butcher shops.

http://dx.doi.org/10.17582/journal.pjz/2017.49.3.861.867: susceptibility testing. Camels.

http://dx.doi.org/10.19045/bspab.2021.100101: *mecA* PCR. Cats and dogs.

12. Also, the authors conclude from their data that there is very limited diversity in S. aureus circulating amongst buffalo farms in this region of Pakistan. Are there animal exchange scenario or workers mobility between the farms studied? This information will be important to further support the conclusion. The authors should consider commenting on how homogenous climate affect the diversity of S. aureus.

-Thank you for this great suggestion. We have add the following information in the:

"This suggests that there is very limited diversity in the S. aureus circulating amongst buffalo farms in this region of Pakistan, which could be due to the close geographic connection between farms and districts in the division, as well as their homogenous climate. With similar environmental factors (such as humidity and temperature) driving selection, similar strain types might prove to be the most fit in each district of this region. Additionally, the farms are small scale and just 5 km apart, meaning the same people could be visiting multiple farms to purchase milk, acting as sources of pathogen transfer. On top of that, the owners of farms in close proximity are often relatives and there can be an exchange of animals between farms, again being a source of pathogen transfer."

Reviewer #2:

The authors have produced a paper that clearly answers the research question posed. Also, the manuscript was comprehensive and well thought out. This work, on the epidemiology and molecular characterisation of S. aureus in bovine mastitis in Pakistan, is a very interesting addition to the literature where globally mastitis has a significant economic impact. It was also interesting to find that the predominant S. aureus CC, found in water buffalo mastitis, was not CC97 or CC151, the most frequently seen CC in cattle mastitis, but the livestock associated strain, CC9, commonly isolated from pigs in Asia.

-Thank you for your appreciation of our manuscript.
