# Supplementary material for: Epidemiology and molecular characterization of Staphylococcus aureus causing bovine mastitis in water buffaloes from the Hazara division of Khyber Pakhtunkhwa, Pakistan
Source: PLoS One. 2022 May 5;17(5):e0268152. doi: 10.1371/journal.pone.0268152 (PMC9071125; doi:10.1371/journal.pone.0268152)
Supplement: Attachment — Submitted filename: Response to reviewers 2.pdf [file pone.0268152.s003.pdf]

PONE-D-22-02764R1 Response to reviewers

Reviewer #1:

The authors addressed all the comments and considered all the suggestions. I have no further comments.

Reviewer #2:

I think that this publication has been well thought out and presented. However, there are a few very minor points.

Line 46-47 states "While numerous studies etc". This statement needs to be referenced.

-several references added

Line 86 an should read and.

-thank you, corrected

Line 94 gentamycin - spelling is gentamicin.

-corrected

Line 96 Please state the company name of the antibiotic disk supplier.

-added

Line 105 Switch times are in seconds.

-added

Line 108 The author should differentiate between antiseptic resistance genes (qac) and antibiotic resistance genes (mup).

-differentiated

Line 126 34.55% SFMT positive but figure for antimicrobial growth on line 127 states 43.55%.

-Sorry, corrected

Line 130 the number 27 should be written as Twenty-seven as it begins the sentence

-amended

line 227-228 Table 5 again the author should differentiate antiseptic and antibiotic resistance genes

-differentiated

Line 360 "however, in one study characterized" would read better as "however, in a study that characterized"

-sorry, the word was missed during final track change review.

Line 422 "With limited data currently available, this become crucial background" This sentence is not clear.

-Changed to: "With limited molecular data currently available, our study provides crucial background information for future researchers, and could help elucidate the epidemiological spread of a pathogen that causes significant economic impact on a major industry in the country."
